# How Medical Students Manage Depression, Anxiety, and Stress: A Cross-Sectional Study

**DOI:** 10.2196/74218

**Published:** 2025-07-07

**Authors:** Jonathan Shaw, Ashley Lai, Sasha Singh, Seung Rim Yoo, Maha Fathali, Laura Stuck, James Hagerty, Van Le, Jisu Shin, Charles Lai, Peter Bota, Aaron Jacobs

**Affiliations:** 1 School of Medicine, California University of Science and Medicine, 1501 Violet St, Colton, CA, 92324, United States, 1 909498003; 2 Medical Education, California University of Science and Medicine, Colton, CA, United States

**Keywords:** medical student, stress, depression, anxiety, coping

## Abstract

Given the established challenging nature of medical school, first- and second-year medical students were surveyed on their feelings of stress alongside their coping mechanisms; the study found a higher level of stress among second-year medical students, indicating a need for support during difficult transition periods.

## Introduction

Due to the intense pressure and stress of the training process, medical students face a higher risk of burnout and depression than the general US population [1]. They must pursue extracurriculars, like research, to be competitive for residency applications. Common stressors include academics, peer-to-peer relationships, and finances [2], which can impact sleep quality, physical and mental health, and academic performance [3]. Stress can lead to burnout, with some even considering leaving medicine [4]. Medical students may cope with stress through healthy habits like exercise or harmful ones like alcohol abuse [5,6].

This study explores how first- and second-year medical students manage and reduce stress, to improve medical student wellness efforts.

## Methods

### Participants and Recruitment

Overall, 120 first-year and 126 second-year students at a California allopathic medical school were contacted via institutional email to complete an anonymous survey. One survey round was sent, with 27 responses collected.

### Measures

The survey comprised 2 demographic questions (school year and gender); 24 multiple-choice questions on stress management; 1 free-response question for any unlisted stress management activities and their frequency; and 21 randomly shuffled questions from the Depression, Anxiety and Stress Scale–21 Items (DASS-21) [7]. The hobbies listed were based on community observation.

### Statistical Analysis

Data were analyzed using IBM SPSS Statistics (version 28.0.1.0). Normality was assessed via the Kolmogorov-Smirnov test. For nonnormally distributed data, a Kruskal-Wallis test (via *k* independent samples) was conducted, with gender and school year as the grouping variables and the range being 1-2. Additionally, Spearman correlations were obtained through SPSS’s bivariate correlation function.

### Ethical Considerations

This study received ethical approval from the California University of Science and Medicine Institutional Review Board (HS-2023‐56) on November 27, 2023. Informed consent was obtained from all participants included in the study for data collection and analyses. Identifying information was not collected. Participants were not compensated.

## Results

The Kolmogorov-Smirnov test found that neither the questions nor the DASS-21 subscores were normally distributed. The Kruskal-Wallis results are presented in Table 1. Spearman correlations showed that school year was positively and moderately correlated with stress (*r*_27_=.699; *P*<.001), anxiety (*r*_27_=.585; *P*<.001), and depression (*r*_27_=.408; *P*=.03).

**Figure 1.**
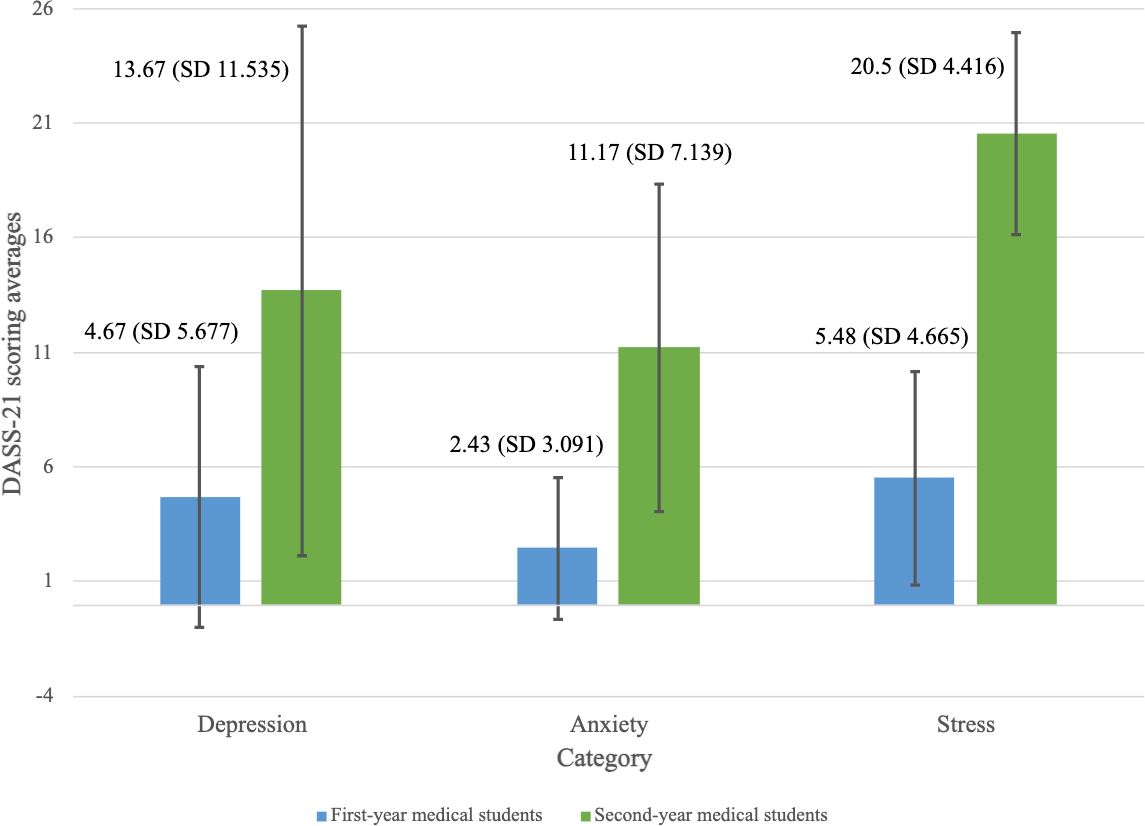
DASS-21 scoring differences across the Depression, Anxiety, and Stress sections between first- and second-year medical students. The DASS-21 can be divided into 3 subsections: Depression, Anxiety, and Stress. Depression thresholds included normal (0‐9), mild (10-13), moderate (14-20), severe (21-27), and extremely severe (28+). Anxiety thresholds included normal (0‐7), mild (8-9), moderate (10-14), severe (15-19), and extremely severe (20+). Stress thresholds included normal (0‐14), mild (15-18), moderate (19-25), severe (26-33), and extremely severe (34+). Averaged DASS-21 scores are displayed, with the bars representing the SD. DASS-21: Depression, Anxiety and Stress Scale–21 Items.

Second-year medical students had mild depression (13.67), with moderate anxiety (11.17) and stress (20.50; Figure 1). In contrast, first-year medical students were in the “normal” range across all categories. This demonstrates that second-year medical students experience higher levels of distress.

**Table 1.** The Kruskal-Wallis results. DASS-21^a^ scoring was conducted by summing the Likert-scale responses (0=did not apply to me at all, 3=applied to me very much or most of the time) for each subscale (Depression, Anxiety, and Stress) and multiplying this sum by 2 to determine the participant severity per subscale. Depression thresholds included normal (0‐9), mild (10-13), moderate (14-20), severe (21-27), and extremely severe (28+). Anxiety thresholds included normal (0‐7), mild (8-9), moderate (10-14), severe (15-19), and extremely severe (20+). Stress thresholds included normal (0‐14), mild (15-18), moderate (19-25), severe (26-33), and extremely severe (34+). All other questions used a Likert scale (0=not at all, 3=nearly every day), asking participants if they had partaken in a stress management behavior within the last 2 weeks. This included a free-response question, which asks participants to fill in any unlisted activities and self-report the frequency of the activity in the same manner as the Likert scale.

Question or topic	Kruskal-Wallis *H* (*df*=1)	*P* value	Group 1, mean (SD)	Group 2, mean (SD)
Read books to relax	4.994	.02	.67 (.900)^b^	.00 (.000)^c^
Talk with friends	5.888	.02	2.28 (.895)^b^	1.44 (.726)^c^
Go out with friends in person	5.081	.02	1.14 (.910)^d^	.20 (.447)^e^
Painting	4.000	.046	.00 (.000)^d^	.75 (1.500)^e^
Find it harder to wind down	12.222	.001	.62 (.590)^d^	2.50 (.837)^e^
Aware of having a dry mouth	10.300	.001	.10 (.301)^d^	1.50 (1.225)^e^
Could not experience positive feelings	7.502	.006	.14 (.359)^d^	1.33 (1.366)^e^
Difficulty breathing	5.234	.02	.10 (.301)^d^	.67 (.816)^e^
Overreacting to situations	6.131	.01	.19 (.402)^d^	1.17 (1.169)^e^
Using a lot of nervous energy	13.211	.001	.57 (.676)^d^	2.33 (.516)^e^
Getting more agitated	6.911	.009	.57 (.870)^d^	2.00 (1.095)^e^
Difficult to relax	9.453	.002	.81 (.873)^d^	2.50 (.837)^e^
Felt downhearted and blue	4.374	.04	.48 (.602)^d^	1.33 (1.033)^e^
Felt close to panic	8.576	.003	.14 (.478)^d^	1.17 (1.169)^e^
Feeling scared without any good reason	5.565	.02	.24 (.539)^d^	1.17 (1.169)^e^
Stress levels (DASS-21)	12.715	.001	5.48 (4.665)^d^	20.50 (4.416)^e^
Anxiety levels (DASS-21)	8.894	.003	2.43 (3.091)^d^	11.17 (7.139)^e^
Depression levels (DASS-21)	4.336	.04	4.67 (5.677)^d^	13.67 (11.535)^e^

a DASS-21: Depression, Anxiety and Stress Scale–21 Items.

b Female group.

c Male group.

d First-year group.

e Second-year group.

## Discussion

Female participants were more likely to read (*H*_1_=4.994; *P*=.02) and talk with friends (*H*_1_=5.888; *P*=.02). This aligns with the existing literature, as women have been shown to read more than men, but this could also be due to our limited sample size [8]. Exercise habits did not significantly differ by school year (*P*=.09) or gender (*P*=.71). Prior studies have shown that exercise reduces burnout and improves quality of life [6], suggesting that medical schools should encourage physical activity, especially for students studying remotely.

First-year medical students were more likely to go out with friends (*H*_1_=5.081; *P*=.02). Second-year medical students had varied schedules, due to rotations or professional development semesters, which at this institution are a dedicated 6-month period for self-directed activities such as research projects or studying for the United States Medical Licensing Examination (USMLE) Step exams. This may limit socialization opportunities compared to first-year medical students, who remain on a shared schedule. On the DASS-21, second-year medical students had moderate stress levels compared to first-year medical students—who had “normal” stress levels (a 15-point difference)—potentially due to clinical rotations, professional development semesters, or Step 1 exam preparation. The 6‐ to 8-week “dedicated” study period is highly stressful, and despite Step 1’s transition to a pass-or-fail grade, concerns remain about its impact on mental health. However, it is important to acknowledge that due to the limited sample size (n=27), it would be difficult to make broad generalizations. Similarly, this raises the question of validity, as students at other medical schools may have different experiences.

These findings highlight the need to focus on increasing social support through events such as mug painting, potlucks, or board game nights. This will help students build a network, better cope with stress, and reduce the negative feelings they experience [9]. Future research should focus on optimizing such interventions not only for second-year medical students but also for third- and fourth-year medical students, who were not included in this study. Future studies should also measure stress management and stress levels longitudinally across cohorts of various institutions so that standardized approaches can be developed, which can further be adjusted to fit the cultures of individual institutions.

## Abbreviations

DASS-21: Depression, Anxiety and Stress Scale–21 Items
USMLE: United States Medical Licensing Examination
